# Identification of a High-Affinity Pyruvate Receptor in *Escherichia coli*

**DOI:** 10.1038/s41598-017-01410-2

**Published:** 2017-05-03

**Authors:** Stefan Behr, Ivica Kristoficova, Michael Witting, Erin J. Breland, Allison R. Eberly, Corinna Sachs, Philippe Schmitt-Kopplin, Maria Hadjifrangiskou, Kirsten Jung

**Affiliations:** 1 Munich Center for Integrated Protein Science (CIPSM) at the Department of Microbiology, Ludwig-Maximilians-Universität München, 82152 Martinsried, Germany; 2 Helmholtz Zentrum München, Deutsches Forschungszentrum für Gesundheit und Umwelt (GmbH), Research Unit Analytical BioGeoChemistry, 85764 Neuherberg, Germany; 3 Departments of Pharmacology, Vanderbilt University Medical Center, Nashville, TN 37232 USA; 4 Departments of Pathology, Microbiology & Immunology, Vanderbilt University Medical Center, Nashville, TN 37232 USA; 5 Departments of Urologic Surgery, Vanderbilt University Medical Center, Nashville, TN 37232 USA

## Abstract

Two-component systems are crucial for signal perception and modulation of bacterial behavior. Nevertheless, to date, very few ligands have been identified that directly interact with histidine kinases. The histidine kinase/response regulator system YehU/YehT of *Escherichia coli* is part of a nutrient-sensing network. Here we demonstrate that this system senses the onset of nutrient limitation in amino acid rich media and responds to extracellular pyruvate. Binding of radiolabeled pyruvate was found for full-length YehU in right-side-out membrane vesicles as well as for a truncated, membrane-integrated variant, confirming that YehU is a high-affinity receptor for extracellular pyruvate. Therefore we propose to rename YehU/YehT as BtsS/BtsR, after “**B**renz**t**rauben**s**äure”, the name given to pyruvic acid when it was first synthesized. The function of BtsS/BtsR was also assessed in a clinically relevant uropathogenic *E*. *coli* strain. Quantitative transcriptional analysis revealed BtsS/BtsR importance during acute and chronic urinary-tract infections.

## Introduction

Exponential growth of bacteria in complex, nutrient-rich media usually ends when at least one nutrient has been used up. We recently reported that the histidine kinase/response regulator system YehU/YehT of *E*. *coli*, belongs to the LytS/LytTR family and presumably plays a role in tuning bacterial exploitation of available carbon sources^1^. Strikingly, the YehU/YehT system is the most widespread representative of its family found in γ-proteobacteria – and many LytS/LytTR-type systems regulate crucial host-specific mechanisms during infection of human or plant hosts by members of this bacterial clade^2^. This system is conserved in non-pathogenic as well as pathogenic *E*. *coli*.

**Figure 1:**
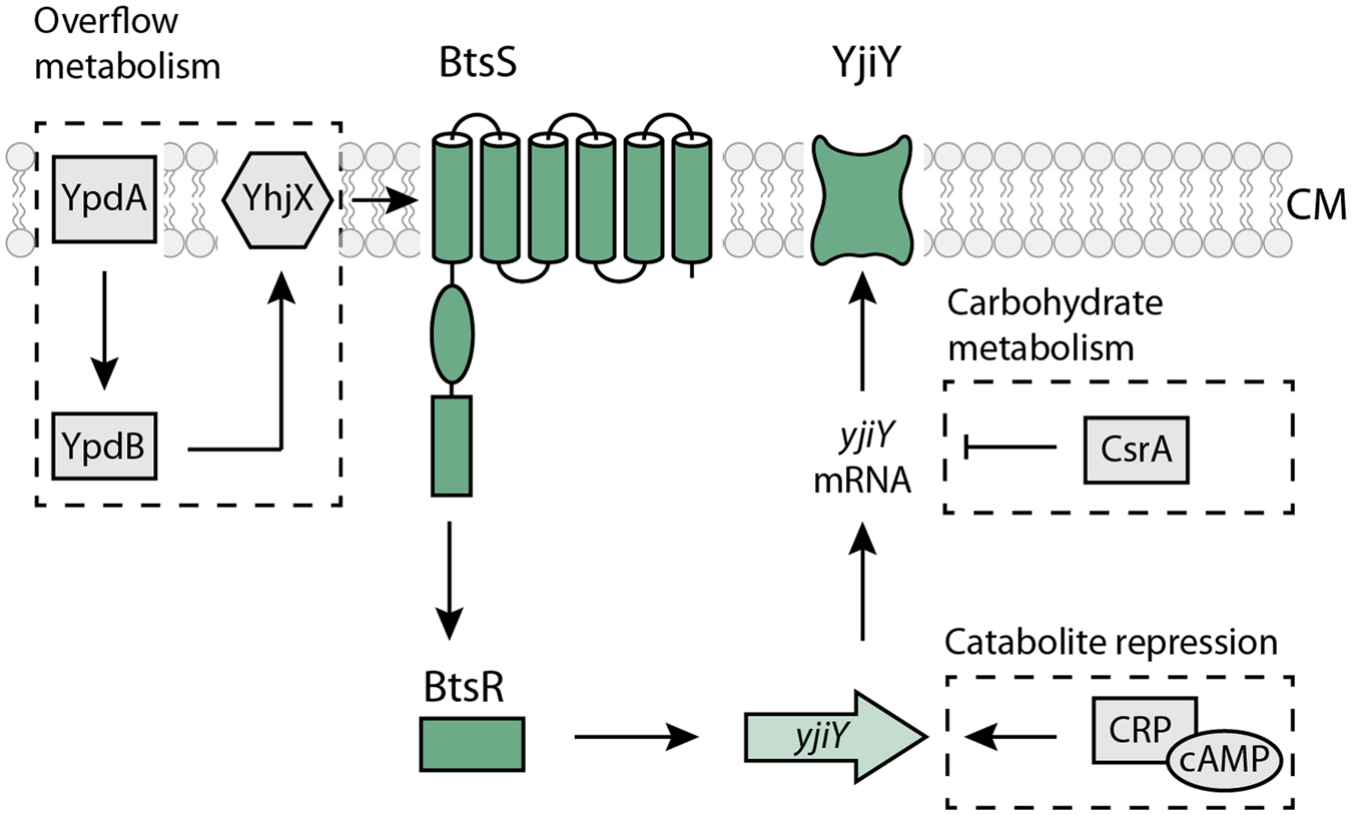
Schematic depiction of the BtsS/BtsR (YehU/YehT) system in *Escherichia coli*. The scheme summarizes the regulatory network associated with signal transduction by the BtsS/BtsR two-component system, the influence of two-component system YpdA/YpdB and the global regulators CsrA and CRP. Membrane proteins are integrated in the cytoplasmic membrane (CM). Activating (↑) and inhibitory (⊥) effects are indicated. See text for details.

Our previous studies on YehU/YehT in *E*. *coli* identified *yjiY* as its sole target gene^3^ (Fig. 1). This gene codes for the putative carbon starvation transporter YjiY, which is homologous (61.1% identity) to CstA^4^ and was found to be expressed in cells that were grown in complex media containing a high content of amino acids, such as LB or CAA (casamino acids), as well as in minimal medium supplemented with certain carbon sources, such as gluconic or glucuronic acid. Studies in *E*. *coli* revealed that YehT-mediated *yjiY* transcription is also regulated by the cAMP/CRP complex^3^ (Fig. 1), and down-regulated in the presence of energetically favorable carbon sources like glucose. Furthermore, YjiY is subject to translational control via the Csr regulatory circuit^5^ (Fig. 1), which synchronizes the output of *E*. *coli* central carbohydrate metabolism (glycolysis versus gluconeogenesis) with YjiY production^1, 6^. Finally, *yjiY* transcription is under positive feedback regulation by a second two-component system, YpdA/YpdB, and its gene product YhjX^1^ (Fig. 1).

Here, we performed a comprehensive *in vivo* characterization of *yjiY* expression in order to identify the primary stimulus sensed by the histidine kinase YehU. We found that the YehU/YehT system responds to depletion of nutrients specifically serine and the concomitant presence of extracellular pyruvate. Biochemical studies revealed that pyruvate binds specifically to the extracellular side of the membrane-spanning domain of YehU. We therefore renamed the system BtsS/BtsR, for “**B**renz**t**rauben**s**äure”, the original name given by Jöns Jakob Berzelius to the compound when he first synthesized pyruvic acid in 1835^7^. Finally, we found that the BtsS/BtsR system of uropathogenic *E*. *coli* may contribute to acute urinary tract infection.

## Results

### Elucidation of the stimulus for BtsS/BtsR (YehU/YehT) by *in vivo yjiY* expression analyses

**Figure 2:**
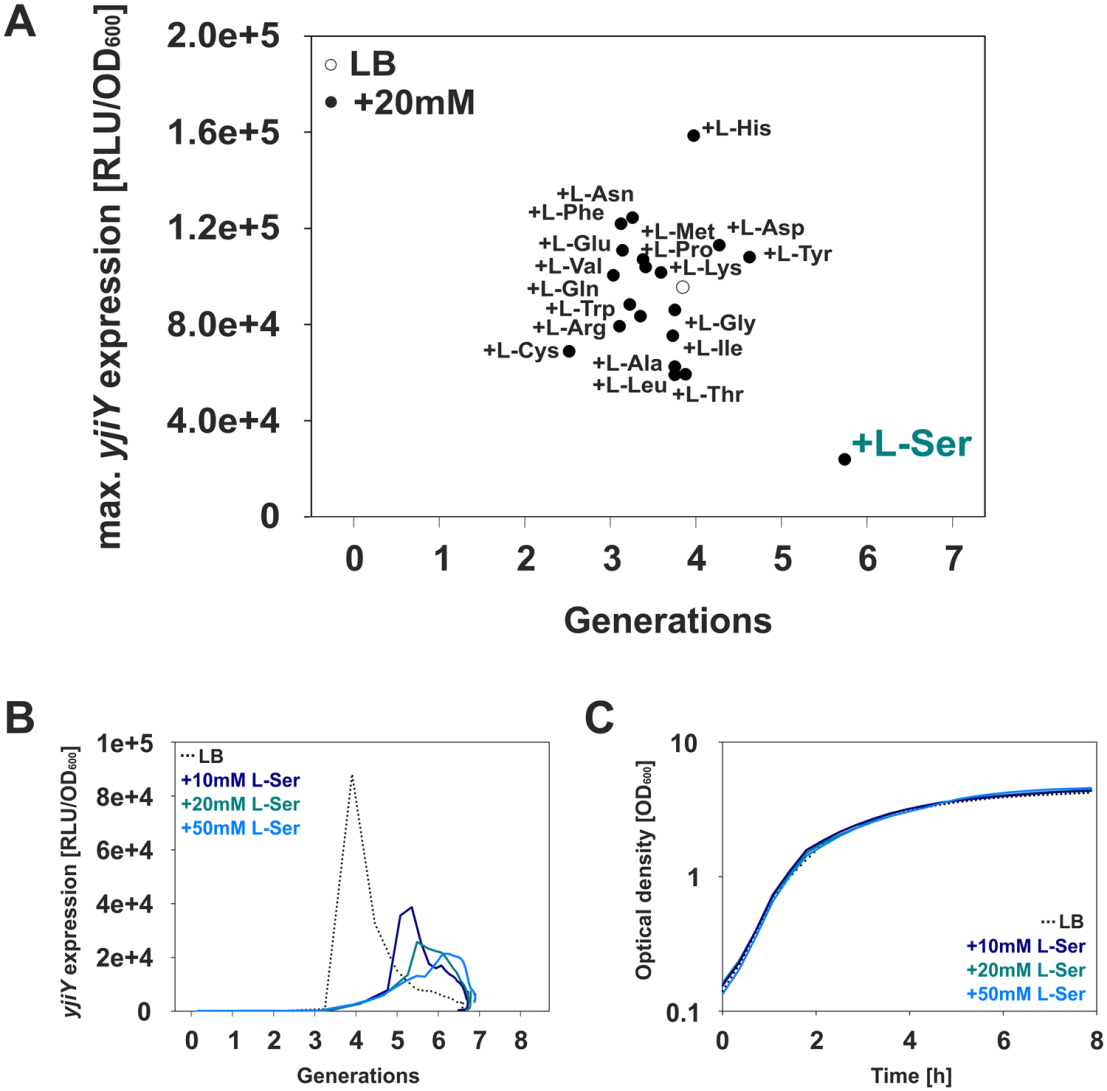
Effects of an excess of individual amino acids on the expression of *yjiY*. (**A**) *E*. *coli* MG1655/pBBR *yjiY*-*lux* was grown in LB medium supplemented with one of the indicated amino acids (at 20 mM), and growth and luminescence were monitored over time. Maximal *yjiY* expression values are depicted. The open circle provides a benchmark and indicates *yjiY* expression in *E*. *coli* grown in LB medium. (**B**) Expression of *yjiY* in LB (dotted line) supplemented with increasing L-serine concentrations. (**C**) Corresponding *E*. *coli* growth curves in LB media supplemented with increasing L-serine concentrations. Experiments were performed at least three times (standard deviation <10%), and results of a representative experiment are shown.

It was previously shown that cultivation of *E*. *coli* in amino acid-rich media leads to activation of the BtsS/BtsR system and transient expression of the target gene *yjiY* in the late-exponential growth phase^3^. To identify a potential quorum sensing-like molecule, we used a combination of chemical fractionation of the medium and measurements of reporter strain activity. For this purpose, we cultivated *E*. *coli* MG1655 Δ*yjiY*/pBBR *yjiY-lux* in M9-minimal medium with gluconic acid as sole carbon source^3^. Shortly before the induction of *yjiY* we removed the cells and fractionated the supernatant by high pressure liquid chromatography. All fractions were analyzed for their potential to induce *yjiY* using reporter strain *E*. *coli* KX1468 pBBR *yjiY-lux* grown in minimal medium and succinate as C-source. After several rounds of fractionation/freeze-drying we found that the fraction with the highest induction potential contained a high concentration of gluconic acid, the initial carbon source (data not shown). This result ruled out the possibility that *E*. *coli* produces and senses a quorum sensing-like molecule. Then we quantified *yjiY* expression as a function of nutrient levels. For this purpose, we cultivated the reporter strain *E*. *coli* MG1655/pBBR *yjiY*-*lux* in LB medium with decreasing amounts of nutrients (1.0x, 0.5x, 0.4x, 0.3x LB, 0.2x LB and 0.1x LB), keeping the osmolarity of the medium constant. The growth rates (µ) of *E*. *coli* cells decreased with the dilution of LB medium, and exponential growth ceased at different time points (Table S1). Strikingly, expression of *yjiY* always began shortly before the onset of stationary phase (Fig. S1), and *E*. *coli* cells grown in 0.1x LB did not express *yjiY*. These results suggested that BtsS/BtsR somehow responds to nutrient limitation. We reasoned that supplying the relevant nutrient(s) in excess should suppress or postpone *yjiY* induction. Therefore, the reporter strain was grown in LB media supplemented with an excess of each individual L-amino acid (Fig. 2A). Particularly, the addition of L-serine delayed the expression of *yjiY* by almost two doubling periods (Fig. 2A). Subsequently, we tested different concentrations of L-serine in the reporter assay and found a concentration-dependent delay in *yjiY* expression, accompanied by a decrease in peak expression levels (Fig. 2B). The addition of serine does not influence the growth of *E*. *coli* and does not delay the onset of stationary phase (Fig. 2C). Although L-serine is not a preferred carbon source for *E*. *coli* and high external concentrations are actually toxic to the organism, it is the first amino acid to be consumed when mixtures of amino acids are available^8^. These data suggest that BtsS/BtsR responds to depletion of nutrients, specifically serine.

### Changes in extracellular serine and pyruvate concentrations during growth of *E*. *coli*

**Figure 3:**
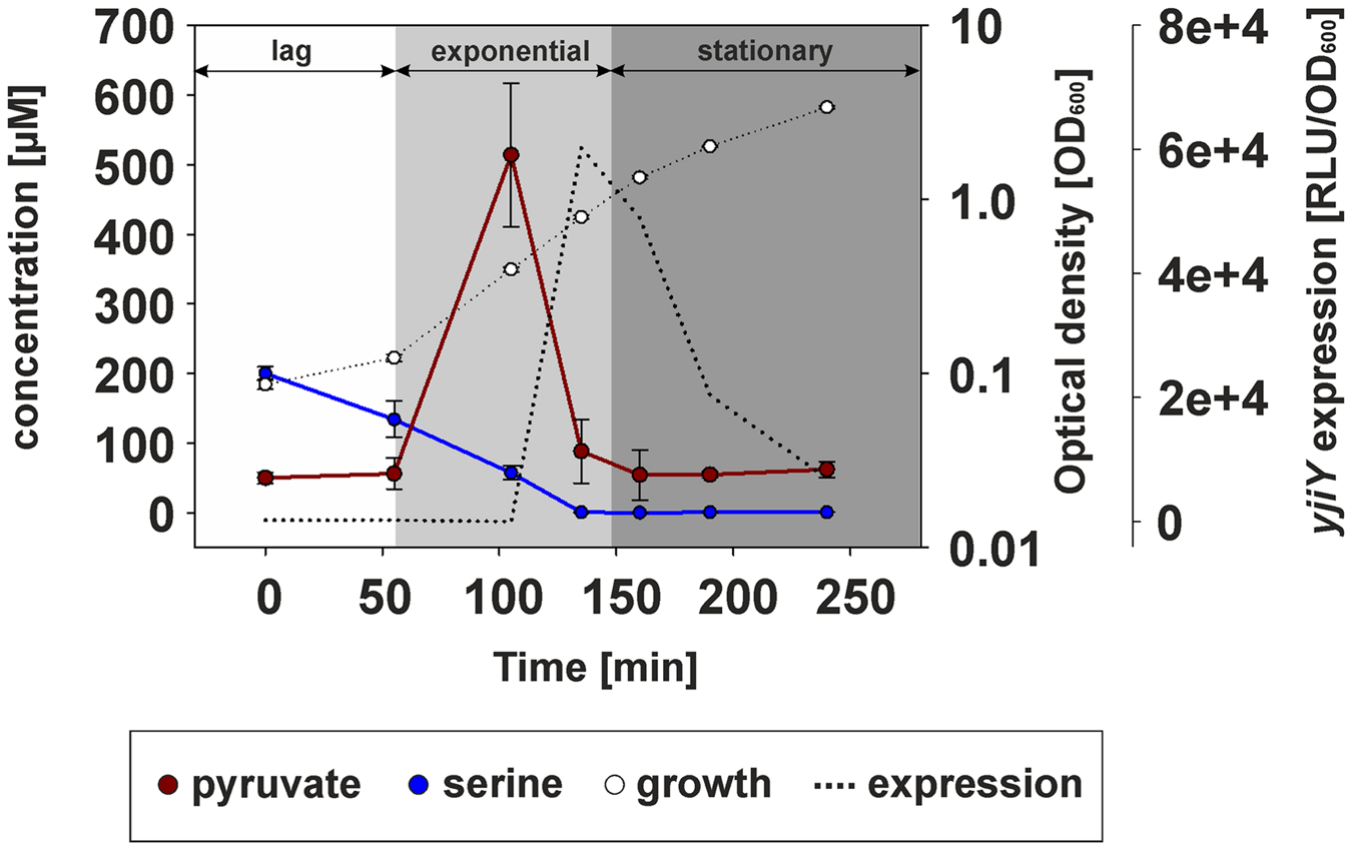
Determination of changes in extracellular concentrations of serine and pyruvate during growth of *E*. *coli*. *E*. *coli* MG1655/pBBR *yjiY*-*lux* was cultivated in LB medium, and growth (OD_600_) and luminescence were monitored. At the times indicated, cells were harvested, and serine and pyruvate levels were quantified by hydrophilic interaction liquid chromatography. All experiments were performed in triplicate, and the error bars indicate the standard deviation of the means. The growth phases of *E*. *coli* are marked as following: lag phase (white), exponential growth (light grey) and stationary phase (dark grey).

When *E*. *coli* is grown in amino acid-rich media, 50.7% of L-serine is converted directly to pyruvate, whereas 36.3% is used for glycine synthesis, 6.5% for cell biomass, and the remainder for other metabolites^9^. Its central role in pyruvate supply provides one explanation for the importance of L-serine in growing *E*. *coli* ^10^. We therefore monitored the changes in extracellular serine and pyruvate concentrations during growth in LB medium, and found that extracellular levels of serine decreased at a constant rate (Fig. 3). The starting concentration of serine in the medium (approximately 200 µM) was completely exhausted after 120 min of growth at the late-exponential growth phase. At the same time, the abundance of extracellular pyruvate peaked (approx. 500 µM) and shortly after *yjiY* expression reached its maximum level (Fig. 3). It was previously shown that the external pyruvate derives from overflow metabolism in *E*. *coli* during growth in amino acid-rich media^11^, which was confirmed by monitoring the intracellular concentrations of serine and pyruvate (Fig. S2).

These data reveal that induction of BtsS-dependent *yjiY* expression coincides with the decline of serine in the medium and an extracellular accumulation of pyruvate (Fig. 3).

### Extracellular pyruvate triggers *yjiY* expression under nutrient limitation

**Figure 4:**
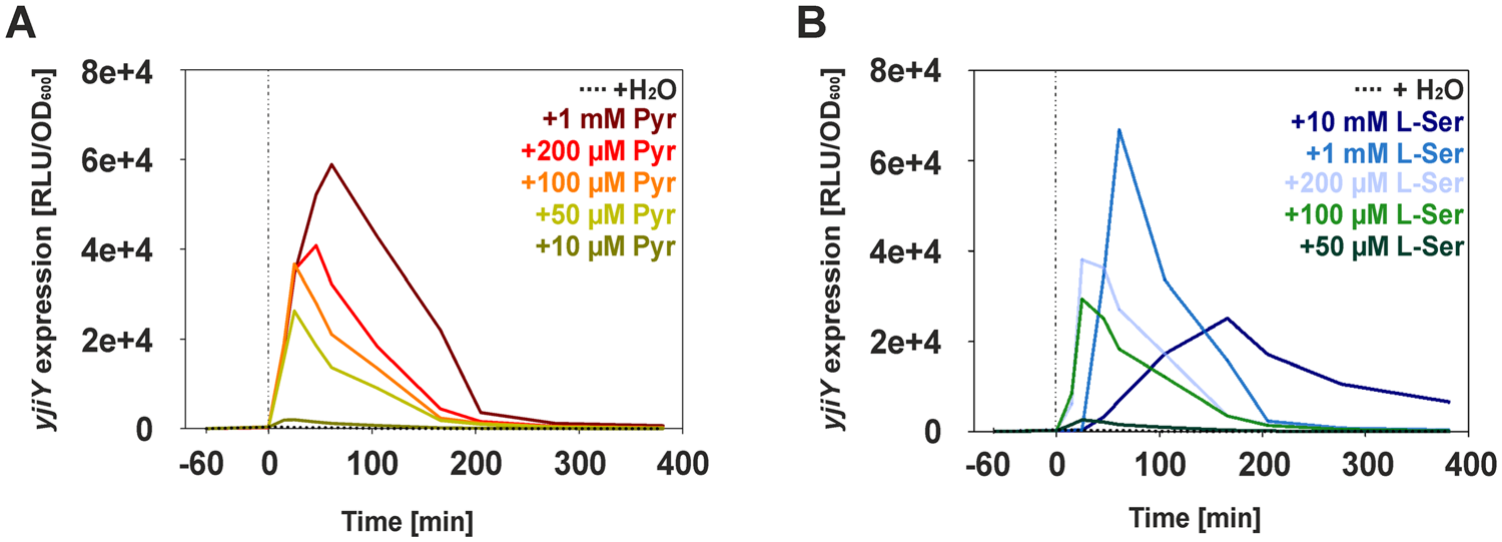
Stimulus-dependent *yjiY* expression under nutrient-limiting conditions. *E*. *coli* MG1655 mutant Δ*yhjX* harboring pBBR *yjiY*-*lux* was cultivated in 0.1x LB medium to establish low nutrient conditions. After 1 h (time point 0), the indicated concentration of pyruvate (**A**), or L-serine (**B**), or the equivalent volume of water was added. Experiments were performed at least three times (standard deviation <10%), and results of a representative experiment are shown.

In the next experiment we tested the influence of serine, pyruvate and related metabolites on *yjiY* expression in *E*. *coli* cells growing in low-nutrient environment. Since *E*. *coli* harbors a second two-component system, YpdA/YpdB, which responds to high concentrations of extracellular pyruvate (the threshold concentration that leads to induction was determined to be 600 µM) and positively regulates the BtsS/BtsR system^11^, we modified our reporter strain by deleting *yhjX*, which is sufficient to interrupt the feedback loop^1^. The resulting strain was then cultivated in 10-fold diluted (0.1x) LB medium for 1 h. At this time point, cells do not induce expression of *yjiY*, but experience soon carbon limitation (Fig. 4A, Fig. S1). However, expression of *yjiY* was rapidly triggered upon addition of pyruvate, and the induction level increased linearly with increasing pyruvate concentration (Fig. 4A). Addition of L-serine also induced *yjiY* expression, but only after a 20-min delay (Fig. 4B). Under these conditions the growth of *E*. *coli* did not differ significantly by addition of pyruvate or serine (Fig. S3). Only supplementation of 10 mM serine prolonged the exponential growth phase. Moreover, higher serine concentrations delayed *yjiY* expression for even longer, and decreased the level of induction attained (Fig. 4B). The threshold concentration of pyruvate required for detectable *yjiY* expression was 10 µM, and that for L-serine 50 µM. None of the other tested compounds (each of the other 19 amino acids, phosphoenolpyruvate, lactate, oxaloacetate, α-ketoglutarate, valeriate, propionate, acetate, malate) were able to induce *yjiY* in this context. These results suggest that extracellular pyruvate acts as a direct stimulus for BtsS/BtsR-mediated *yjiY* expression, whereas delayed *yjiY* induction in response to L-serine may depend on uptake of the amino acid, its conversion to pyruvate and excretion of the pyruvate into the culture medium.

### The sensor histidine kinase BtsS binds pyruvate with high affinity

**Figure 5:**
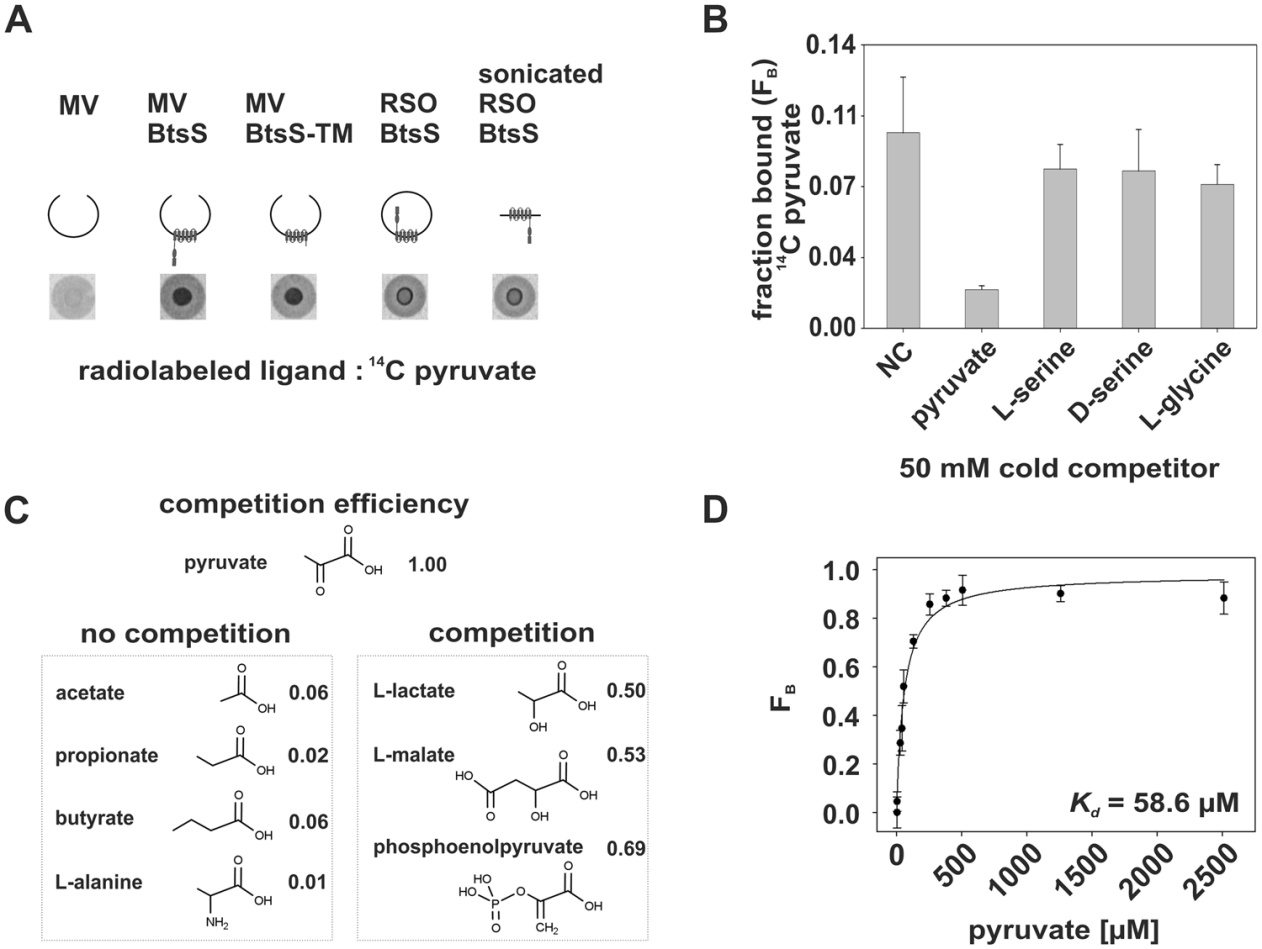
Analysis of the interaction of BtsS with selected ligands by DRaCALA. (**A**) A mixture of membrane vesicles (MV) or right-side-out vesicles (RSO) enriched with the corresponding proteins (indicated by graphical representations) and radiolabeled ^14^C pyruvate (5 µM) is dropped onto a nitrocellulose membrane, and ligand migration via capillary action is analyzed. (**B**) Competition assays. Binding of radiolabeled pyruvate (5 µM) to BtsS in MVs was analyzed in the presence of various unlabeled competitors (each 50 mM). NC, no competitor. (**C**) Relative efficiency of competition by various carboxylic acids. Binding of radiolabeled pyruvate (5 µM) to BtsS in MVs was analyzed in the presence of various carboxylic acids (each 50 mM). The efficiency of competition by cold pyruvate was set to 1.00, and the effect of the indicated compounds was calculated accordingly. (**D**) Determination of the dissociation constant (*K*_*d*_) for pyruvate to BtsS using DRaCALA. For each reaction radiolabeled pyruvate was used at 5 µM. Normalized F_B_ values [F_B_ = (F_B(NC)_ − F_B(pyr)_)/F_B(NC)_, see Methods for details] were plotted as function of the pyruvate concentration. The best-fit line was determined by nonlinear regression using the equation y = B_max_ * x/(*K*_*d*_ + x).

BtsS cannot be autophosphorylated, possibly owing to a defective ATP-binding site within the G1 box^3^ (data not shown). Therefore, we used the method of differential radial capillary action of ligand assays (DRaCALA)^12^ to determine whether BtsS physically interacts with pyruvate as ligand. The technique is based on the ability of proteins that have been immobilized on a nitrocellulose membrane to bind a radiolabeled ligand, whereas unbound ligands undergo radial diffusion. DRaCALA allows rapid detection of both the total ligand and the ligand sequestered by proteins. The fraction of ligand bound to the protein, defined as F_B_, is calculated from the signal intensity of the area with protein (inner circle) and the total signal intensity of the area (outer circle)^12^. For the DRaCALA we used unsealed membrane vesicles prepared from *E*. *coli* cells overproducing BtsS (MV BtsS) and calculated an F_B_ value of >0.15 (Fig. 5A). Control membrane vesicles (MV, lacking overproduced BtsS) were used and the low value of F_B_ (0.05) reflected only minor, non-specific binding. Therefore this assay was judged to be suitable for membrane vesicles, and it clearly indicated binding of radiolabeled pyruvate to BtsS. We also tested ^3^H-serine binding to BtsS in membrane vesicles using the DRaCALA technique. However, we only observed unspecific binding (data not shown).

In addition, we tested MVs harboring a membrane-integrated truncated variant of BtsS (MV BtsS-TM) lacking its soluble domains, and found that this truncated sensor also binds pyruvate (F_B_ > 0.15). Furthermore, we prepared sealed right-side-out vesicles (RSO BtsS), in which membrane proteins retain their native orientation^13^. BtsS in these vesicles also showed pyruvate binding, and no significant change in binding was observed when these vesicles were fragmented by sonication and had a random orientation (sonicated RSO BtsS) (F_B_ values of each 0.18) (Fig. 5A). These data reveal that pyruvate binds to the external side of the membrane-spanning domain of BtsS.

The specificity of pyruvate binding to BtsS was then addressed by using a competition assay (Fig. 5B), in which several competitors were added in excess to the reaction mixture. Only unlabeled (“cold”) pyruvate was able to prevent binding of radiolabeled pyruvate. L-serine, D-serine or glycine did not interfere with pyruvate binding to BtsS, suggesting that pyruvate binds specifically to BtsS.

Next we investigated the effects of varying the length and side-chain charge on ligand binding by BtsS. Using competition assays, we found that acetate, propionate and butyrate all failed to compete with pyruvate for binding, indicating the importance of the C-α keto group of pyruvate (Fig. 5C). The influence of polarity at the C-α position was then tested by addition of lactate and malate, which contain C-α hydroxyl groups. These molecules were able to decrease pyruvate binding by about 50% suggesting that a negative charge seems to be recognized. It should be noted that these compounds were tested in a 10^4^-fold excess over pyruvate. Phosphoenolpyruvate, which harbors a phosphoryl group at the C-α position, also competed with pyruvate, reducing binding by approximately 50%. In contrast, a positive charge at the C-α position in the form of the amino group in alanine had no effect, reducing pyruvate binding by 1% (Fig. 5C). Notably, none of these compounds were able to induce *yjiY* expression *in vivo*, which emphasizes the specificity of BtsS for pyruvate. The dissociation constant (*K*_*d*_) for pyruvate binding to the histidine kinase was found to be 58.6 ± 8.8 µM (Fig. 5D).

To our knowledge this is the first application of DRaCALA to measure the interaction between a ligand and a membrane-embedded protein. Moreover, these assays unambiguously demonstrated that pyruvate binds specifically and with high affinity to BtsS.

### BtsS/BtsR importance during urinary tract infection

To understand the potential implications of BtsS/BtsR for pathogenesis, we therefore turned our attention to uropathogenic *E*. *coli* (UPEC) that colonize a nutritionally demanding environment – the mammalian bladder. However, responses to nutritional stress are of the utmost importance for the survival of both commensal and pathogenic bacteria within a given host.

**Figure 6:**
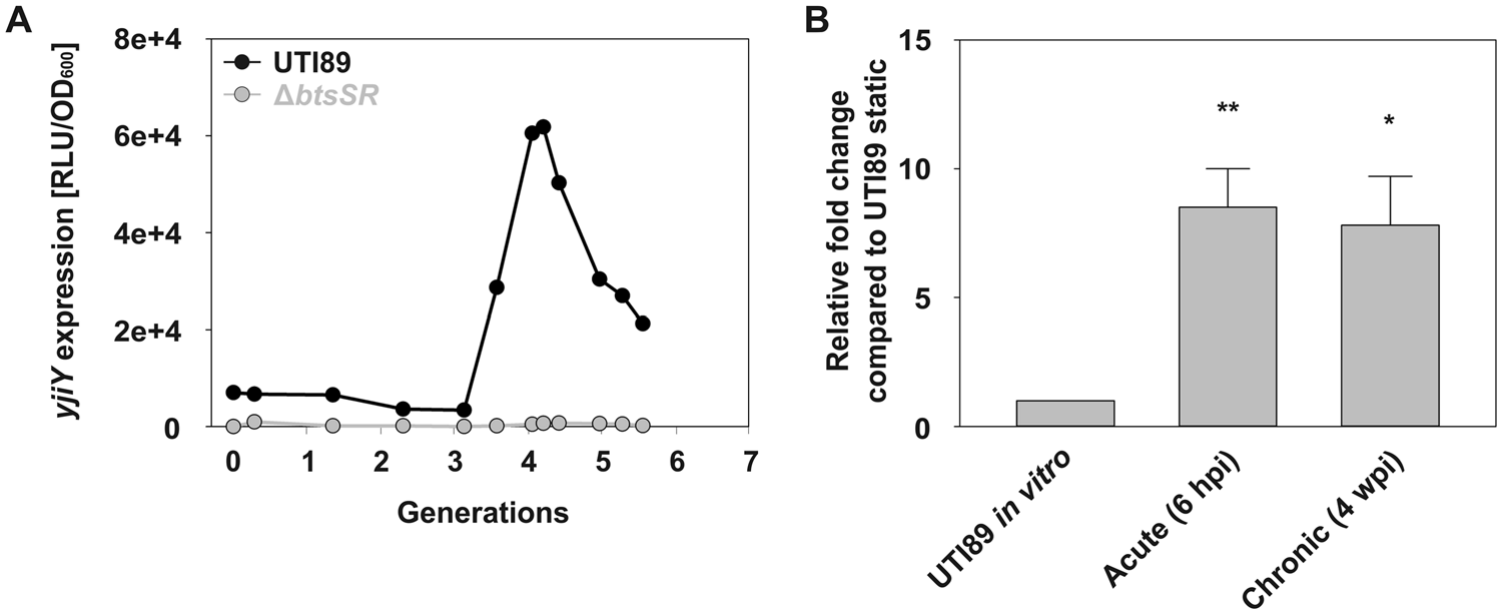
BtsS/BtsR importance during urinary tract infection. (**A**) The plot indicates the activity of the pBBR *yjiY*-*lux* reporter fusion in WT UTI89 and the isogenic *btsS*/*btsR* mutant during growth in LB, and depicts a representative of at least 6 biological replicates. (**B**) Change in the level of the *yjiY* transcript during acute and chronic phases of UTI relative to that measured in samples incubated *in vitro* without agitation. Data shown are the mean values of samples extracted from three different mice per time point. RNA was extracted from dissected bladders for each time point and enriched for bacterial RNA by depleting ribosomal and mammalian RNA as described in Materials and Methods. Profiling was performed using an *yjiY*-specific probe. Expression was normalized to that of the housekeeping gene *gyrB* and compared to expression in cDNA samples corresponding to the starting inoculum (static *in vitro* culture, prepared as described in Materials and methods). Relative fold change was measured using the method described by Pfaffl *et al*.^54^ (hpi, hours post infection; wpi, weeks post infection).

UPEC strains are the primary causative agent of urinary-tract infections (UTIs) worldwide, accounting for over 85% of all reported episodes^14^. In the bladder, bacteria adhere to the apical surface of the epithelium and are internalized, before replicating to form biofilm-like pods within host bladder cells^15, 16^. Subsequent to this transient intracellular cascade, adverse outcomes such as chronic colonization can ensue as a result of an aberrant host immune response^17^. Previous studies have demonstrated that successful UTI requires aerobic respiration and amino acid utilization^18–20^. To investigate the significance of BtsS/BtsR in bladder infection, we first created a *btsS*/*btsR* mutant in the UPEC cystitis isolate UTI89. Using the same reporter fusion, we demonstrated that this strain, unlike the wild-type parental UTI strain, failed to induce transient expression of *yjiY* (Fig. 6A). Both pyruvate and L-serine are present in human and murine urine, and levels are elevated in diabetic populations^21–23^. Given that BtsS/BtsR responds directly to extracellular pyruvate levels and indirectly to L-serine, we asked whether BtsS/BtsR is active in the bladder lumen during acute and chronic UTI.

Mice were infected with the cystitis isolate UTI89 and RNA samples were extracted from bladder homogenates at 6 h post infection (coinciding with the acute stage of UTI), and at 2 weeks post infection from mice that went on to develop chronic cystitis. At this stage, the majority of bacteria are found on the bladder epithelial surface in the form of an extracellular biofilm^17^. Subsequent TaqMan-based qPCR analysis compared the expression of *yjiY* in the corresponding cDNA samples to the expression of *yjiY* in the starting bacterial inoculum. Our results demonstrated robust expression of *yjiY* in both the acute and chronic stages of infection from at least three separate mice per time-point (Fig. 6B). Taken together, these data suggest that, in the murine UTI model BtsS/BtsR responds to serine/pyruvate fluctuations and could play a role in promoting the infection process.

## Discussion

Although numerous studies continue to demonstrate the importance of histidine kinase-mediated signal transduction in bacterial physiology, the natural ligands have been identified for very few histidine kinases^24–26^. This study presents compelling evidence demonstrating that the histidine kinase BtsS is a high-affinity receptor for extracellular pyruvate. Induction of the BtsS/BtsR target gene *yjiY*, which codes for a “carbon starvation” CstA-like transporter, correlates with the depletion of serine in complex medium, explaining the observation that BtsS/BtsR senses low serine. At the same time, while serine is being depleted from LB medium, *E*. *coli* excretes a substantial amount of pyruvate from overflow metabolism into the medium. Shortly afterwards, the concentration of extracellular pyruvate returns to its basal level (Fig. 3 and ref. 11). In previous studies, we demonstrated activation of a different system, YpdA/YpdB in response to high levels of extracellular pyruvate^11^ and showed that induction of YpdA/YpdB enhances BtsS/BtsR activation^1^. The current work provides a comprehensive characterization of how BtsS/BtsR directly responds to extracellular pyruvate levels and fine-tunes the metabolic fitness of *E*. *coli* under low-nutrient conditions. Sequence comparison between YpdA and YehU did not identify putative amino acids involved in pyruvate binding. In non-pathogenic *E*. *coli*, each of these signaling systems induces the expression of exactly one gene, which codes for a transporter. One (YhjX) belongs to the major facilitator superfamily, and is assumed to be a low-affinity carboxylate antiporter^27^. The other one (YjiY) belongs to the CstA-like transporters, which are characterized by an unusually large number of transmembrane helices (17 in the case of YjiY). Neither transporter has been characterized thus far. It is hypothesized that both are involved in nutrient uptake, but with different affinities. The interconnectivity between the high-affinity pyruvate signaling system BtsS/BtsR with the putative low-affinity pyruvate signaling system YpdA/YpdB by a positive and a negative feedback loop^1^ would provide *E*. *coli* with a network that could tailor pyruvate uptake in each individual cell according to its availability. Switching between low- and high-affinity phosphate transporters based on the needs of the individual cell was recently demonstrated for *S*. *cerevisiae* ^28^, and seems to be a widely distributed strategy for the maintenance of nutrient homeostasis as stocks of essential nutrients decline^29^.

Although nutrient sensing is crucial for host-microbe and microbe-microbe interactions, the majority of studies in non-pathogenic *E*. *coli* strains have focused on metabolic engineering, aiming to understand how processes such as elevated intracellular pyruvate levels affect metabolite distribution^30, 31^ or how central mutations trigger/alter metabolic fluxes^32–34^. In recent years, technological advances have permitted detailed analyses of *in vivo* metabolism^35^, revealing its complexity and its influence on virulence and pathogenesis^36^. These studies have shown pyruvate to be one of the major factors connecting cellular metabolism to cell division^37^. Moreover, pyruvate levels are thought to reflect the quantitative relationship between carbon and nitrogen availability in the cell, and affect amino acid biosynthesis^38^.

Investigations of how metabolic decisions determine pathogen fitness within host niches are increasingly uncovering opportunities for the development of robust and pathogen-specific drugs. Different *E*. *coli* pathotypes cause various clinical syndromes, depending on their genetic makeup and expression patterns. They obviously differ extensively from each other and from commensal *E*. *coli*. For example, uropathogenic and enterohemorragic *E*. *coli* differ significantly in genome content, and employ different strategies to infect their niches - the bladder and the gut, respectively. Very recent work has pointed to the need for aerobic respiration and amino acid utilization for optimal colonization of the urinary tract by UPEC^18–20^. Pyruvate is a crucial molecule required for fueling the aerobic arm of the tricarboxylic acid cycle, which is used by UPEC during infection^18, 19, 39^. Thus, it is reasonable to postulate that UPEC has the capacity to sense and respond to pyruvate fluctuations within the urinary tract. Here we demonstrate that BtsS/BtsR mediates *yjiY* expression during infection by a clinically relevant UPEC isolate, which strongly suggests that BtsS/BtsR constitutes part of the metabolic circuitry engaged during UTI. Thus understanding how UPEC can sense the metabolic signature of the bladder lumen, the kidney and the urothelial cell cytosol (in which they form intracellular bacterial communities) will be vital for a complete understanding of pathogen strategies and the design of more effective therapeutics. However, the study in *Salmonella enterica* Serovar Typhi and Typhimurium showed no detectable effect on the ability of *yehUT* mutant strain to invade cultured epithelial cells or induce colitis in a murine model^40^.

In summary, this study has uncovered a signal transduction network that responds with exquisite sensitivity to extracellular pyruvate levels and fine-tunes carbon utilization in *E*. *coli* strains. Furthermore we provide direct evidence for receptor-ligand interactions between BtsS and pyruvate, and demonstrate that BtsS/BtsR mediates *yjiY* expression during UTI. Future studies will focus on understanding the contribution of this system to pathogenic *E*. *coli* intra-host fitness and dissecting molecular determinants that drive the fine-tuning of bacterial fitness in response to external metabolic cues.

## Methods

### Strains, plasmids, and oligonucleotides

In this study we used the *E*. *coli* strains MG1655^41^, *E*. *coli* MG1655 Δ*yhjX* ^1^, *E*. *coli* MG1655 Δ*yjiY* ^1^, BL21(DE3)^42^, *E*. *coli* KX1468^43^ and the cystitis isolate UTI89^44^. Plasmids used in this study include the transcriptional promoter-luciferase fusion construct for P_*yjiY*_ activity (pBBR *yjiY*-*lux*)^3^. For protein production we used the arabinose-inducible expression vectors pBAD24^45^, pBAD24-*yehU* ^3^ and pBAD24-*yehU*-TM (YehU (BtsS) amino acids 1–205). DNA fragments for plasmid construction were amplified from genomic DNA by PCR. Scarless deletion of *btsS*/*btsR* genes in UTI89 was performed using the method published by Murphy and Campellone^46^, and verified via PCR with oligonucleotides flanking the deleted loci. All oligonucleotide sequences are available on request.

### Molecular biological techniques

Plasmid and genomic DNAs were isolated using a HiYield plasmid minikit (Suedlaborbedarf) and a DNeasy blood and tissue kit (Qiagen), respectively. DNA fragments were purified from agarose gels using a HiYield PCR cleanup and gel extraction kit (Suedlaborbedarf). Q5 DNA polymerase (New England BioLabs) was used according to the supplier’s instructions. Restriction enzymes and other DNA-modifying enzymes were also purchased from New England BioLabs and used according to the manufacturer’s directions.

### Growth conditions

All strains were grown overnight in LB medium. After inoculation, bacteria were routinely grown in LB, LB supplemented with the indicated amino acids or diluted LB medium [containing 1% (w/v) NaCl] under agitation (200 rpm) at 37 °C. For solid medium, 1.5% (w/v) agar was added. Where appropriate, media were supplemented with antibiotics (ampicillin sodium salt, 100 µg/ml; kanamycin sulfate, 50 µg/ml; gentamicin sulfate, 50 µg/ml). For infection studies in mice, UTI89 was inoculated into 10 ml of LB medium and grown without shaking at 37 °C for 24 h. This culture was used to seed a fresh flask with 10 ml of a 1:1000 dilution, which was incubated for another 24 h to maximize expression of type 1 pili, as previously described^47^. The growth phases of *E*. *coli* marked in graphs were according to the definition in ref. 48.

### *In vivo* expression studies

*In vivo* expression of *yjiY* was quantified by means of luciferase-based reporter-gene assays, using bacteria that had been transformed with plasmid pBBR *yjiY-lux*. Cells from an overnight culture were transferred to fresh medium to give a starting optical density at 600 nm (OD_600_) of 0.05, and incubated under aerobic growth conditions at 37 °C while OD_600_ and luminescence were continuously monitored. The optical density was determined in a microplate reader (Tecan Sunrise) at 600 nm. Luminescence levels were determined in a luminescence reader (Centro LB960; Berthold Technology) for 0.1 s and are reported as relative light units (RLU; counts s^−1^).

### Identification of quorum sensing-like molecule

The cells of *E*. *coli* MG1655 Δ*yjiY*/pBBR *yjiY-lux* were cultivated in M9-minimal medium supplemented with 20 mM gluconic acid and OD_600_ and luminescence were constantly monitored. Cells were harvested shortly before, at and after the induction of *yjiY*. The supernatant was sterile filtrated (Stericup®, Filter Unit Millipore Express® PLUS(PES), 0.22 μm and separated by high pressure liquid chromatography (HPLC) using column C18-Hypersil Gold, with the gradient 1–100% (v/v) acetonitrile in 40 min. Fractions were collected, concentrated and used in reporter strain *E*. *coli* KX1468^43^/pBBr *yjiY-lux* grown in M9-minimal medium supplemented with 20 mM succinate. The fraction with the highest induction potential was then analyzed via UPLC-UHR-ToF-MS using a Waters XBridge Amide column.

### Production of BtsS-6His in membrane vesicles

*E*. *coli* BL21(DE3) cells were transformed with the vector pBAD24-*yehU* ^3^, which codes for BtsS-6His, and grown in LB medium at 37 °C to an OD_600_ of 0.5. Recombinant gene expression was induced by addition of 0.2% (w/v) arabinose. After 3 h of further incubation, cells were harvested by centrifugation, disrupted and fractionated. At each step, the pellet was resuspended in buffer consisting of MES/NaOH (pH 6), 10% (v/v) glycerol and 10 mM MgCl_2_. To produce BtsS-6His in right-side-out vesicles, cells were cultivated as described before. After harvesting the cells by centrifugation, lysozyme (50 µg/ml) and EDTA (10 mM) were added for 45 min at room temperature to remove the cell wall. After low-speed centrifugation, the spheroplasts were resuspended in buffer consisting of 100 mM MES/NaOH (pH 6), 30% (w/v) sucrose, 20 mM MgSO_4_, DNase and RNase, and then diluted 100-fold into pre-warmed buffer (100 mM MES/NaOH (pH 6)) to trigger osmotic lysis. After centrifugation, the pellet was resuspended in 100 mM MES/NaOH (pH 6), 10% (v/v) glycerol and 10 mM MgCl_2_. Formation of spheroplasts and right-side-out vesicles was monitored under the microscope. His-tagged BtsS was detected by Western blot analysis with an anti-His antibody (Qiagen) and an alkaline phosphatase-conjugated anti-mouse antibody as the secondary antibody.

### Protein-ligand interactions

Protein-ligand interactions were analyzed via DRaCALA, a method established by Roelofs *et al*.^12^. Membrane vesicles enriched for overproduced BtsS-6His were mixed with 5 µM radiolabeled (3-^14^C) pyruvic acid sodium salt (55 mCi mmol^−1^, Biotrend) or radiolabeled (^3^H) L-serine (11.0 Ci mmol^−1^, Hartmann Analytics) and incubated for 5 min at room temperature. Triplicate 5-µl aliquots were transferred to a nitrocellulose membrane, dried and visualized by a PhosphorImager. Quantification of radioactive signal was done with the software ImageQuant. The fraction bound to protein was calculated according to the signal intensities using an equation defined earlier: F_B_ = (I_inner_ − I_background_)/I_total_^12^. For the evaluation the corresponding cold ligand (50 mM) was added to the reaction mixture and incubated for further 3 min. To estimate the *K*_*d*_ value, increasing concentrations of cold pyruvate (0 µM to 2.5 mM) were used. In this case the F_B_ value was normalized as follows: F_B_ = (F_B(NC)_ − F_B(pyr)_)/F_B(NC)_. Here, F_B(NC)_ is calculated from the sample with pure ^14^C-labeled pyruvate, and F_B(pyr)_ are all values for mixtures containing ^14^C-labeled pyruvate together with increasing concentrations of unlabeled pyruvate. The amount of ^14^C-pyruvate was kept constant in all assays.

### Extraction and determination of extra- and intracellular metabolites

The reporter strain *E*. *coli* MG1655/pBBR *yjiY*-*lux* was cultivated in LB medium, and OD_600_ and luminescence were constantly monitored. At selected time points, cells were harvested and the supernatants were saved. Cells were washed with 10% (v/v) glycerol, and subsequently cell pellets and supernatants were analyzed via hydrophilic interaction liquid chromatography (HILIC). Acetonitrile (ACN), methanol (MeOH), ammonium acetate and ammonium hydroxide (all of LC-MS grade) were obtained from Sigma-Aldrich (Sigma-Aldrich GmbH). Water was purified using a Merck Millipore Integral water purification system to a resistance of 18 MΩ and TOC < 5 ppb. Sodium pyruvate and serine were also obtained from Sigma-Aldrich and dissolved in water at a concentration of 100 mM and further diluted with ACN. Cell pellets were extracted with 200 µl ice-cold water/MeOH (50/50, v/v). Samples were vortexed vigorously and lysed in an ice-cold sonic bath for 15 min. After centrifugation at 20,000 × *g* and 4 °C for 10 min, supernatants were transferred to autosampler vials.

Pyruvate and serine were quantified using a modified version of the method published by Yuan *et al*.^49^. Separation was achieved using a Waters XBridge Amide column (3.5 µm, 100 mm × 4.6 mm ID) and an ACN/water gradient using a Waters Acquity UPLC system coupled to a Bruker maXis UHR-ToF-MS (Bruker Daltonics). Eluent A consisted of 5% (v/v) ACN, 95% (v/v) water, 20 mM ammonium hydroxide, 20 mM ammonium acetate, pH 9.0 and eluent B was pure ACN.

Metabolites were eluted with the following gradient: After 3 min of 85% B, a linear decrease to 2% B over 9 min, isocratic hold for 3 min and return to initial conditions for 1 min and a 7-min re-equilibration time. Sample (5 µl) was injected via partial loop injection.

For quantification a calibration curve was generated from different concentrations of standard compounds (0 µM, 0.5 µM, 1.0 µM, 2.5 µM, 5 µM, 10 µM, 25 µM, 50 µM, 100 µM, 250 µM). If the concentration of a sample was beyond the range of the calibration curve, it was appropriately diluted with water/MeOH. Quantification was performed with Bruker Quant Analysis.

### Murine infections

A cohort (15) of 7- to 8-week old female C3H/HeN mice was infected with strain UTI89 via transurethral catheterization as described in Hung *et al*.^50^. Five mice were sacrificed at 6 h after infection, marking the acute stage of UTI in this murine model^51^. Bladders were excised for CFU enumeration and RNA extraction. The remaining 10 mice were monitored for chronic infection using longitudinal urinalysis, as previously described^52^. Mice that consistently shed more than 100,000 CFUs/ml of urine were separated from the rest of the cohort, and flagged as chronically infected. These mice were sacrificed at 4 weeks post infection and bladders were obtained for CFU enumeration and RNA extraction.

To ensure the proper and humane treatment of animals, all animal studies were carried out in strict accordance with the recommendations in the Guide for the Care and Use of Laboratory Animals of the National Institutes of Health, and the Vanderbilt University Medical Center’s Institutional Animal Care and Use Committee (IACUC), who approved all protocols. Statistical analyses were performed using two-tailed Mann-Whitney t-test.

### RNA extraction, cDNA synthesis and qPCR

RNA extraction and bacterial RNA enrichment were performed as described by Conover *et al*.^47^. DNase treatment, reverse transcription, and real-time quantitative PCR were done as described by Guckes *et al*.^53^. qPCR analysis was carried out with three concentrations of cDNA (50 ng, 25 ng, 12.5 ng), each in triplicate for each sample, and levels of *gyrB* (DNA gyrase) were used for normalization. The following primers (Integrated DNA Technologies) were used for amplification: *yjiY*_Fwd (5′-GGCACGACGCCGAAACT-3′), *yjiY*_Rev (5′-GCCGTAGCCGATGAAACG-3′), *gyrB*_L (5′-GATGCGCGTGAAGGCCTGAATG-3′), *gyrB*_R (5′-CACGGGCACGGGCAGCATC-3′). The following probes (Applied Biosystems) were used for quantification; *yjiY* (5′FAM-TGGCTAATGAAACCGACGC-MGBNFQ-3′); *gyrB* (5′VIC-ACGAACTGCTG-GCGGA-MGBNFQ-3′).

## Electronic supplementary material

Supplementary information

## References

[1] Behr S, Fried L, Jung K (2014). Identification of a novel nutrient-sensing histidine kinase/response regulator network in *Escherichia coli*. J. Bacteriol.

[2] Galperin MY (2008). Telling bacteria: do not LytTR. Structure.

[3] Kraxenberger T, Fried L, Behr S, Jung K (2012). First insights into the unexplored two-component system YehU/YehT in. Escherichia coli. J. Bacteriol..

[4] Schultz JE, Matin A (1991). Molecular and functional characterization of a carbon starvation gene of *Escherichia coli*. J. Mol. Biol..

[5] Romeo T, Vakulskas CA, Babitzke P (2013). Post-transcriptional regulation on a global scale: form and function of Csr/Rsm systems. Environ. Microbiol..

[6] Sabnis NA, Yang H, Romeo T (1995). Pleiotropic regulation of central carbohydrate metabolism in *Escherichia coli* via the gene *csrA*. J. Biol. Chem.

[7] Berzelius J (1835). Ueber die Destillationsproducte der Traubensäure. Ann. Phys.

[8] Prüss B, Nelms JM, Park C, Wolfe AJ (1994). Mutations in NADH: ubiquinone oxidoreductase of *Escherichia coli* affect growth on mixed amino acids. J. Bacteriol.

[9] Selvarasu S (2009). Characterizing *Escherichia coli* DH5α growth and metabolism in a complex medium using genome‐scale flux analysis. Biotechnol. Bioeng..

[10] Polzin S (2013). Growth media simulating ileal and colonic environments affect the intracellular proteome and carbon fluxes of enterohemorrhagic *Escherichia coli* O157: H7 strain EDL933. Appl. Environ. Microbiol..

[11] Fried L, Behr S, Jung K (2013). Identification of a target gene and activating stimulus for the YpdA/YpdB histidine kinase/response regulator system in *Escherichia coli*. J. Bacteriol.

[12] Roelofs KG, Wang J, Sintim HO, Lee VT (2011). Differential radial capillary action of ligand assay for high-throughput detection of protein-metabolite interactions. Proc. Natl. Acad. Sci. USA.

[13] Kaback H (1971). Bacterial Membranes. Methods Enzymol..

[14] Foxman B (2010). The epidemiology of urinary tract infection. Nat. Rev. Urol.

[15] Mulvey MA (1998). Induction and evasion of host defenses by type 1-piliated uropathogenic *Escherichia coli*. Science.

[16] Anderson GG (2003). Intracellular bacterial biofilm-like pods in urinary tract infections. Science.

[17] Hannan TJ (2012). Host–pathogen checkpoints and population bottlenecks in persistent and intracellular uropathogenic *Escherichia coli* bladder infection. FEMS Microbiol. Rev..

[18] Alteri CJ, Smith SN, Mobley HL (2009). Fitness of *Escherichia coli* during urinary tract infection requires gluconeogenesis and the TCA cycle. PLoS Pathog..

[19] Hadjifrangiskou M (2011). A central metabolic circuit controlled by QseC in pathogenic *Escherichia coli*. Mol. Microbiol.

[20] Floyd KA (2015). Adhesive fiber stratification in uropathogenic *Escherichia coli* biofilms unveils oxygen-mediated control of type 1 pili. PLoS Pathog..

[21] Stec DF (2015). Alterations of urinary metabolite profile in model diabetic nephropathy. Biochem. Biophys. Res. Com.

[22] Bouatra S (2013). The human urine metabolome. PloS One.

[23] Kaur P (2013). Quantitative metabolomic and lipidomic profiling reveals aberrant amino acid metabolism in type 2 diabetes. Mol. BioSyst..

[24] Reinelt S, Hofmann E, Gerharz T, Bott M, Madden DR (2003). The structure of the periplasmic ligand-binding domain of the sensor kinase CitA reveals the first extracellular PAS domain. J. Biol. Chem..

[25] Cheung J, Hendrickson WA (2009). Structural analysis of ligand stimulation of the histidine kinase NarX. Structure.

[26] Gudipaty SA, McEvoy MM (2014). The histidine kinase CusS senses silver ions through direct binding by its sensor domain. Biochim. Biophys. Acta.

[27] Pao SS, Paulsen IT, Saier MH (1998). Major facilitator superfamily. Microbiol. Mol. Biol. Rev..

[28] Wykoff DD, Rizvi AH, Raser JM, Margolin B, O’Shea EK (2007). Positive feedback regulates switching of phosphate transporters in *S*. *cerevisiae*. Mol. Cell.

[29] Levy S, Kafri M, Carmi M, Barkai N (2011). The competitive advantage of a dual-transporter system. Science.

[30] Yang Y-T, Bennett GN, San K-Y (2001). The effects of feed and intracellular pyruvate levels on the redistribution of metabolic fluxes in *Escherichia coli*. Metab. Eng..

[31] Sauer U, Eikmanns BJ (2005). The PEP-pyruvate-oxaloacetate node as the switch point for carbon flux distribution in bacteria. FEMS Microbiol. Rev..

[32] Fischer E, Sauer U (2003). Metabolic flux profiling of *Escherichia coli* mutants in central carbon metabolism using GC‐MS. Eur. J. Biochem..

[33] Wang Q, Ou MS, Kim Y, Ingram L, Shanmugam K (2010). Metabolic flux control at the pyruvate node in an anaerobic *Escherichia coli* strain with an active pyruvate dehydrogenase. Appl. Environ. Microbiol..

[34] Murarka A, Clomburg JM, Gonzalez R (2010). Metabolic flux analysis of wild-type *Escherichia coli* and mutants deficient in pyruvate-dissimilating enzymes during the fermentative metabolism of glucuronate. Microbiology.

[35] Eylert E (2008). Carbon metabolism of *Listeria monocytogenes* growing inside macrophages. Mol. Microbiol.

[36] Eisenreich W, Dandekar T, Heesemann J, Goebel W (2010). Carbon metabolism of intracellular bacterial pathogens and possible links to virulence. Nat. Rev. Microbiol..

[37] Göhler A-K (2011). More than just a metabolic regulator - elucidation and validation of new targets of PdhR in *Escherichia coli*. BMC Syst. Biol..

[38] Chubukov V, Gerosa L, Kochanowski K, Sauer U (2014). Coordination of microbial metabolism. Nat. Rev. Microbiol..

[39] Floyd KA (2016). The UbiI (VisC) aerobic ubiquinone synthase is required for expression of type 1 pili, biofilm formation, and pathogenesis in uropathogenic *Escherichia coli*. J. Bacteriol.

[40] Wong VK (2013). Characterization of the *yehUT* Two-Component Regulatory System of *Salmonella enterica* Serovar Typhi and Typhimurium. PLoS One.

[41] Blattner F (1997). The complete genome sequence of *Escherichia coli* K-12. Science.

[42] Studier FW, Moffatt BA (1986). Use of bacteriophage T7 RNA polymerase to direct selective high-level expression of cloned genes. J. Mol. Biol..

[43] Xavier KB, Bassler BL (2005). Regulation *of uptake and processing of the quorum-sensing autoinducer AI-2* in Escherichia coli. J. Bacteriol..

[44] Schilling JD, Mulvey MA, Vincent CD, Lorenz RG, Hultgren SJ (2001). Bacterial invasion augments epithelial cytokine responses to *Escherichia coli* through a lipopolysaccharide-dependent mechanism. J. Immunol..

[45] Guzman L, Belin D, Carson M, Beckwith J (1995). Tight regulation, modulation, and high-level expression by vectors containing the arabinose P_BAD_ promoter. J. Bacteriol..

[46] Murphy KC, Campellone KG (2003). Lambda Red-mediated recombinogenic engineering of enterohemorrhagic and enteropathogenic *Escherichia coli*. BMC Mol. Biol..

[47] Conover MS (2016). Metabolic Requirements of *Escherichia coli* in Intracellular Bacterial Communities during Urinary Tract Infection Pathogenesis. mBio.

[48] Pörtner, R. Grundlagen der Bioverfahrenstechnik. In Antranikian, G. (Ed.) *Angewandte Mikrobiologie* Springer-Verlag, Berlin, Heidelberg p. 240 (2006)

[49] Yuan M, Breitkopf SB, Yang X, Asara JM (2012). A positive/negative ion-switching, targeted mass spectrometry-based metabolomics platform for bodily fluids, cells, and fresh and fixed tissue. Nat. Protoc..

[50] Hung C-S, Dodson KW, Hultgren SJ (2009). A murine model of urinary tract infection. Nat. Protoc..

[51] Justice SS (2004). Differentiation and developmental pathways of uropathogenic *Escherichia coli* in urinary tract pathogenesis. Proc. Natl. Acad. Sci. USA.

[52] Hannan TJ, Mysorekar IU, Hung CS, Isaacson-Schmid ML, Hultgren SJ (2010). Early severe inflammatory responses to uropathogenic *Escherichia coli* predispose to chronic and recurrent urinary tract infection. PLoS Pathog..

[53] Guckes KR (2013). Strong cross-system interactions drive the activation of the QseB response regulator in the absence of its cognate sensor. Proc. Natl. Acad. Sci. USA.

[54] Pfaffl MW (2001). A new mathematical model for relative quantification in real-time RT–PCR. Nucleic Acids Res.

