# Supplementary material for: Identification of a High-Affinity Pyruvate Receptor in Escherichia coli
Source: Sci Rep. 2017 May 3;7:1388. doi: 10.1038/s41598-017-01410-2 (PMC5431176; doi:10.1038/s41598-017-01410-2)
Supplement: Supplementary file 1 — Supplementary information [file 41598_2017_1410_MOESM1_ESM.pdf]

Supplementary Material:

Identification of a High-Affinity Pyruvate Receptor in *Escherichia coli*

Stefan Behr[a], Ivica Kristoficova[a], Michael Witting[b], Erin J. Breland[c], Allison R. Eberly[d], Corinna Sachs[a], Philippe Schmitt-Kopplin[b], Maria Hadjifrangiskou[d,e], Kirsten Jung[a*]

[a] Munich Center for Integrated Protein Science (CIPSM) at the Department of Microbiology, Ludwig-Maximilians-Universität München, 82152 Martinsried, Germany

[b] Helmholtz Zentrum München, Deutsches Forschungszentrum für Gesundheit und Umwelt (GmbH), Research Unit Analytical BioGeoChemistry, 85764 Neuherberg, Germany

[c] Departments of Pharmacology, Vanderbilt University Medical Center, Nashville, TN 37232, USA

[d,e] Departments of Pathology, Microbiology & Immunology [d] and Urologic Surgery [e], Vanderbilt University Medical Center, Nashville, TN 37232, USA

* To whom correspondence should be addressed: Dr. Kirsten Jung, Ludwig-Maximilians-Universität München, Department Biologie I, Bereich Mikrobiologie, Großhaderner Str. 2-4, 82152 Martinsried, Germany. Phone: +49-89-2180-74500; Fax: +49-89-2180-74520; E-mail: jung@lmu.de

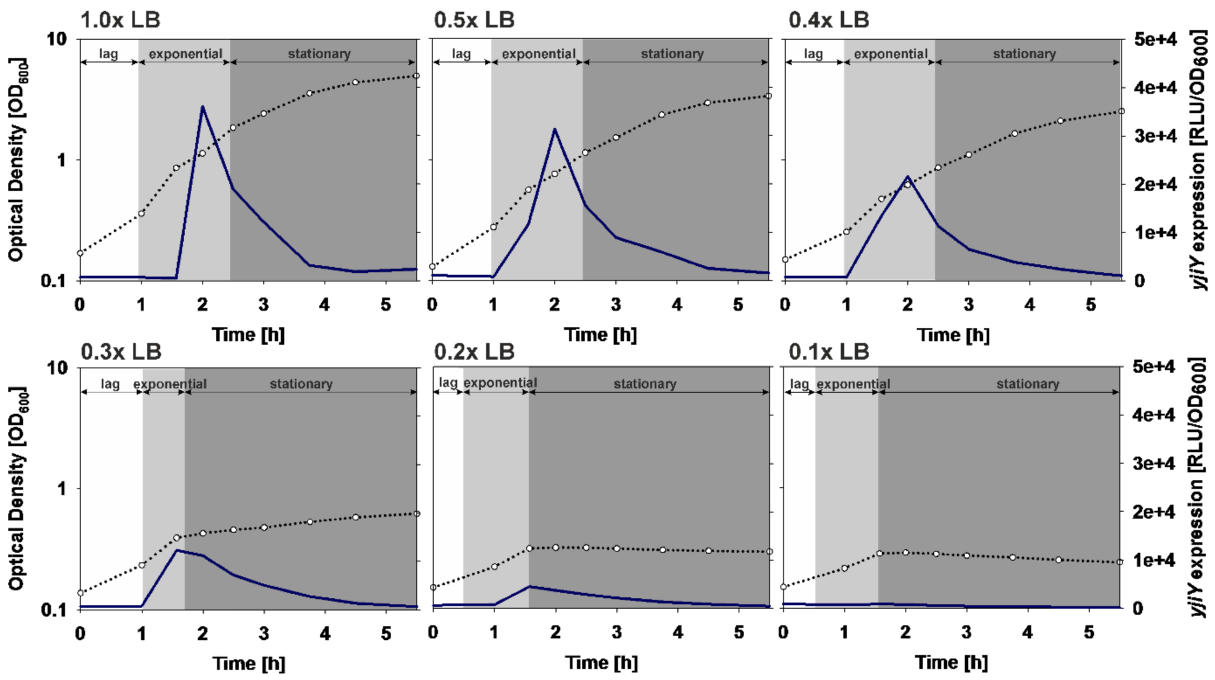

Fig. S1. Characterization of *yjiY* expression in different concentrations of LB medium. *Escherichia coli* MG1655 / pBBR *yjiY*-lux was cultivated under aerobic conditions and growth and luminescence were measured over time. Expression of *yjiY* over growth of *E. coli* cells in 1.0x, 0.5x, 0.4x, 0.3x, 0.2x and 0.1x diluted LB. The growth phases of *E. coli* are marked as following: lag phase (white), exponential growth (light grey) and stationary phase (dark grey).

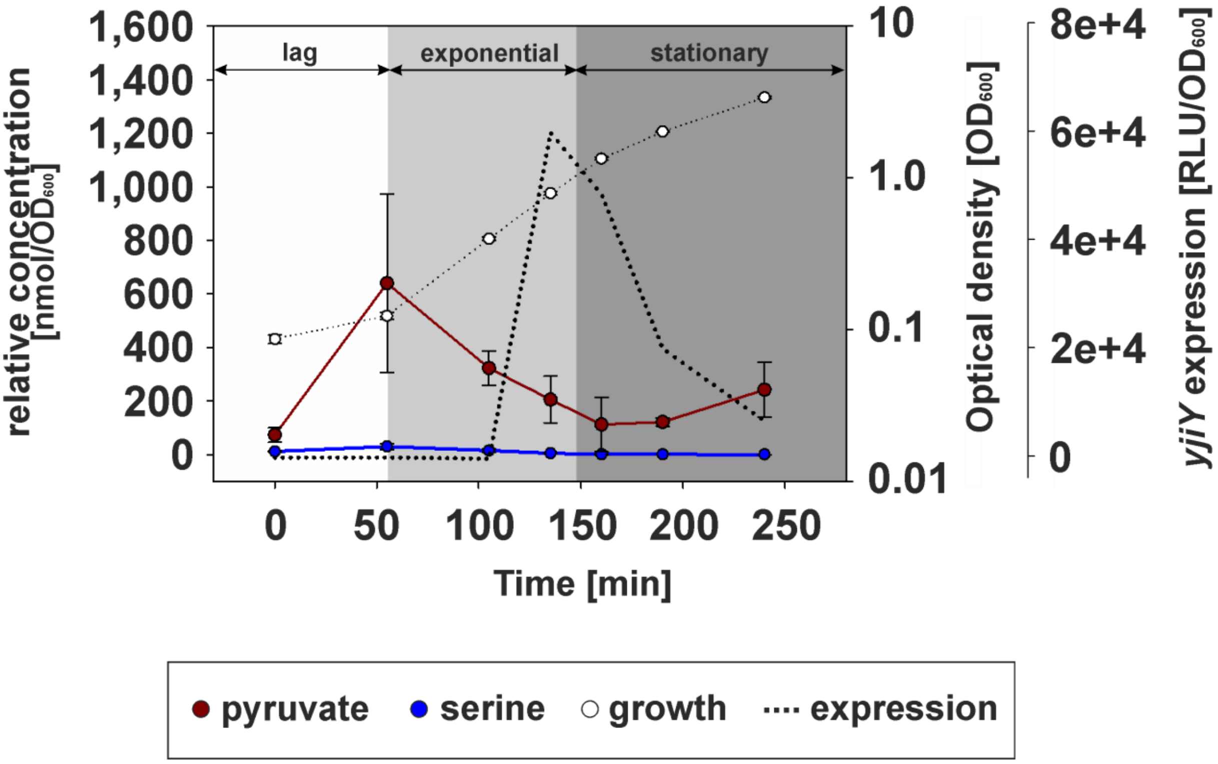

Fig. S2. Determination of changes in intracellular concentrations of serine and pyruvate during growth of *E. coli*. *E. coli* MG1655/pBBR *yjiY-lux* was cultivated in LB medium, and growth (OD600) and luminescence were monitored. At the times indicated, cells were harvested, and serine and pyruvate levels were quantified by hydrophilic interaction liquid chromatography. All experiments were performed in triplicate, and the error bars indicate the standard deviation of the means. The growth phases of *E. coli* are marked as following: lag phase (white), exponential growth (light grey) and stationary phase (dark grey).

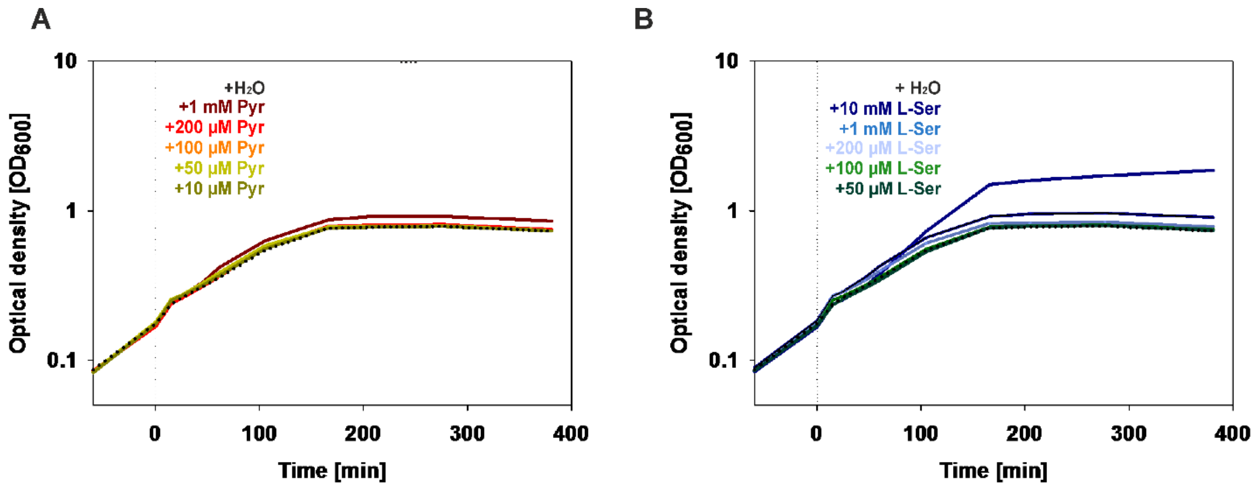

Fig. S3. Corresponding *E. coli* growth curves under nutrient-limiting conditions. *E. coli* MG1655 mutant Δ*yhjX* harboring pBBR *yjiY-lux* was cultivated in 0.1x LB medium. After 1 h (time point 0), the indicated concentration of pyruvate (A), or L-serine (B), or the equivalent volume of water was added. Growth was monitored over time.

Table S1. Summary of growth and *yjiY* expression data from *E. coli* cells grown in LB and diluted LB medium Growth rates for each time point (t(x)) were determined with μ = [ln (OD600 (x)) - ln (OD600 (x-1))] / [t(x) - t(x-1)].

Medium	Growth rate μ	max OD_{600}	OD_{600} at max. *yjiY* expression	max. *yjiY* expression [RLU/OD_{600}]
1.0x LB	1.27 h^{-1}	4.96	1.13	36,009
0.5x LB	1.18 h^{-1}	3.36	0.76	31,272
0.4x LB	0.95 h^{-1}	2.53	0.62	21,629
0.3x LB	1.05 h^{-1}	0.62	0.39	12,315
0.2x LB	0.75 h^{-1}	0.3	0.28	4,536
0.1x LB	0.63 h^{-1}	0.24	not detectable	no expression
